# Moving “Forward” in *Plasmodium* Genetics through a Transposon-Based Approach

**DOI:** 10.1155/2012/829210

**Published:** 2012-05-08

**Authors:** Bharath Balu

**Affiliations:** Tropical Disease Research Program, Center for Infectious Disease and Biodefense Research, SRI International, Harrisonburg, VA 22802, USA

## Abstract

The genome sequence of the human malaria parasite, *Plasmodium falciparum*, was released almost a decade ago. A majority of the *Plasmodium* genome, however, remains annotated to code for hypothetical proteins with unknown functions. The introduction of forward genetics has provided novel means to gain a better understanding of gene functions and their associated phenotypes in *Plasmodium*. Even with certain limitations, the technique has already shown significant promise to increase our understanding of parasite biology needed for rationalized drug and vaccine design. Further improvements to the mutagenesis technique and the design of novel genetic screens should lead us to some exciting discoveries about the critical weaknesses of *Plasmodium*, and greatly aid in the development of new disease intervention strategies.

## 1. Introduction

Malaria is a serious global health problem causing clinical illness in hundreds of millions of people and killing around a million, each year [1]. Intervention strategies to control the disease have been largely ineffective due to increased parasite drug resistance, ineffective vector control measures, and inadequate knowledge about parasite biology to identify new drug and vaccine targets. The need to discover critical weaknesses in the parasite that could be exploited in designing novel antiparasitic strategies is greater than ever. Since the release of the *Plasmodium* genome sequence, several large-scale functional studies have advanced our overall knowledge tremendously about parasite biology [2–6]. However, in spite of such enormous efforts, almost 50% of the genome remains annotated to code for hypothetical proteins in all *Plasmodium* species [7]. It is imperative to understand the functions and essentiality of these hypothetical proteins to facilitate the identification of novel drug or vaccine targets.

## 2. Difficulties in *Plasmodium* Genetics

The first and foremost obstacle in functional characterization of the *Plasmodium* genome is our limited ability to genetically manipulate the parasite [8]. Out of all the *Plasmodium* species that cause human malaria, only the blood stages of *P. falciparum* can be cultured *in vitro* effectively and is the only life cycle stage amenable to transfection with exogenous DNA. While electroporation is most effective in transfecting *P. falciparum* [9, 10], the transfection efficiency is very low, in the range of 10^−6^ [11]. The rodent malaria parasite, *P. berghei*, can be transfected with much higher efficiency and, therefore, has been used more extensively in targeted gene knockout studies [12]. Furthermore, transfections in *P. falciparum* are limited to circular plasmid DNA and the parasite's ability to maintain these plasmids as episomes makes it difficult to isolate the rare genomic integration events [13, 14]. Lastly, the parasite's haploid genome in the blood stages limits manipulation and identification of genes essential for parasite development.

## 3. Reverse Genetics versus Forward Genetics

**Figure 1:**
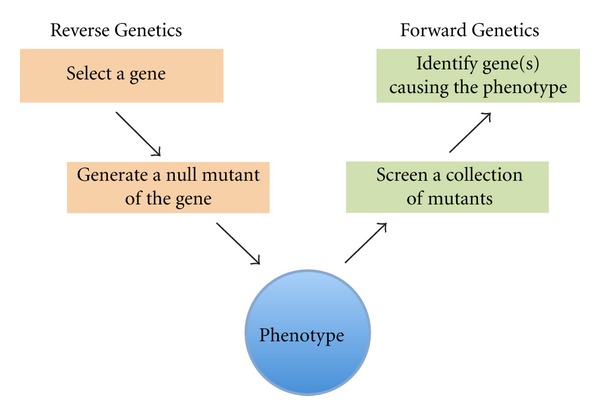
A schematic representation of forward and reverse genetic approaches for genetic association of phenotypes. Reverse genetics starts with the selection of gene of interest and culminates with the phenotypic analysis of its disruption. Forward genetics starts by selecting a phenotype of interest and ends in identifying gene(s) responsible for that phenotype.

There are two possible approaches to functional characterization of a genome: (1) a reverse genetic approach, where a gene of interest is disrupted and the resulting phenotype is studied and (2) a forward genetic approach, where a phenotype of interest is first chosen and a pool of randomly generated mutants is subjected to a screen for the phenotype of interest (Figure 1). Both these approaches have been equally effective, depending on the organism studied [15–18]. Functional characterization of the yeast genome is a great example of a successful reverse genetic approach [19]. A genome-wide knockout study has been feasible in yeast because of the ease of introducing exogenous DNA and the high efficiency of homologous recombination, and a large amount of phenotypic information is currently available for each gene knockout [20]. In higher organisms such as the fruit fly, forward genetic screens using transposon-mediated insertional mutagenesis have been preferred and have provided great information regarding gene functions [21–23]. A recent branch of forward genetics using small molecules has been very promising in many organisms, including *Plasmodium* [24–28]. Such chemical genetic approaches directly inhibit protein function and are often reversible. This paper will focus specifically on a transposon-based forward genetic approach and its applications in *Plasmodium*.

## 4. Transposon-Mediated Mutagenesis in *Plasmodium*

### 4.1. Rationale

Reverse genetic studies in *Plasmodium*, especially in the most lethal human malaria parasite *P. falciparum*, have been hampered by low efficiencies of transfection and homologous recombination [8]. Although a few medium-scale gene knockout approaches have been attempted in *P. falciparum*, they were extremely tedious, labor intensive, and fall much short of the genome-wide knockouts needed for rationalized drug and vaccine design [29, 30]. Forward genetic studies might offer a quicker and more efficient way to characterize the *Plasmodium* genome. The transposition event, which includes finding one target sequence from hundreds of thousands in the genome, should be much more efficient than homologous recombination at a single locus in the genome. Performing genetic screens might thus be faster and more effective in identifying genes involved in critical biological processes of the parasite.

The* piggyBac* transposable element has been used extensively for insertional mutagenesis in numerous invertebrate and vertebrate species [31–35]. The ability to transmobilize *piggyBac*, through separate expression of the *piggyBac* transposase, has immensely contributed to the wide adaptability of the system to multiple organisms [36]. *piggybac*'s ability to more randomly insert into the genome, compared to other transposable elements, increases the likelihood of achieving genome-wide insertions and supports its application in insertional mutagenesis studies [35]. The target site for *piggyBac* insertion is TTAA and more than 300,000 TTAA target sites can be found in the AT-rich *Plasmodium* genome, providing a platform for genome-wide mutagenesis [37].

### 4.2. Methodology

**Figure 2:**
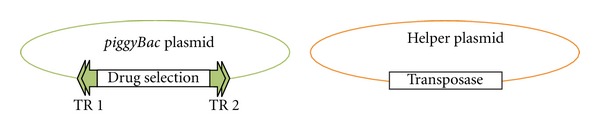
Basic plasmid designs for *piggyBac* mutagenesis in *Plasmodium*. The donor plasmid consists of a *Plasmodium* drug selection cassette flanked by the terminal repeats (TR) of *piggyBac*. The helper plasmid provides the *piggyBac* transposase, which mediates the transposition event into the *Plasmodium* genome upon cotransfection with the donor plasmid.

The *piggyBac* system was first successfully adapted to *P. falciparum* [38] and has recently been extended to *P. berghei* [39]. The basic design for *piggyBac* insertional mutagenesis in *Plasmodium* uses a two-plasmid approach. The first plasmid consists of a *Plasmodium* drug selection cassette flanked by the *piggyBac *5′ and 3′ terminal repeats, and the second plasmid drives the expression of the *piggyBac* transposase, required for the enzymatic cleavage and genomic insertion of the *piggyBac* terminal repeats (Figure 2). The transposase plasmid lacks a drug-selection cassette and is lost in a few generations, thereby preventing continuous movement of *piggyBac*. In *P. berghei*, a transgenic line containing the *piggyBac* transposase in the genome was also shown to be very effective in insertional mutagenesis and remobilization of previously inserted *piggyBac* element [39]. The precise insertion and excision mechanisms of *piggyBac* leave no footprints behind following remobilization, allowing phenotype rescue of any knockout [34].

### 4.3. Efficiency of *piggyBac* Mutagenesis in *Plasmodium*

*piggyBac* insertions into the genome can be obtained within 3-4 weeks in *P. falciparum* compared to 6–12 months needed to obtain homologous recombination events, and 1–10 insertions can be obtained per transfection depending on the conditions used [38]. Genomic insertions of *piggyBac* can be obtained in much higher numbers in *P. berghei* due to its increased transfection efficiency. Analyses of approximately 200 *piggyBac* insertion sites in *P. falciparum* and 120 in *P. berghei* show an almost equal distribution in the coding and noncoding regions of the genome, as predicted by the equal distribution of TTAA target sites in both coding and noncoding regions [39, 40]. This is an important finding considering the higher AT-richness in the noncoding regions of the genome. While a slight bias was observed for insertions into the 5′ untranslated regions (UTRs) of genes in the initial study in *P. falciparum*, this bias was most likely due to the higher growth rate of clones with insertions in UTRs compared to those with insertions in coding sequences, in a mixed population of transformed parasites, and this bias for 5′ UTRs was not observed in experiments where the transfection was followed by immediate cloning of parasites (Balu and Adams, unpublished data).

 There appears to be no striking bias for insertion into any particular chromosome or chromosomal region. Furthermore, *piggyBac* inserts into genes of all functional categories and shows no significant preference for genes expressed in the transformed blood stages. An equivalent number of *piggyBac* insertions were observed in genes exclusively expressed in other parasite life cycle stages, indicating the accessibility to these genes in the parasite's blood stages. Such observations confirm the randomness of *piggyBac* insertions and demonstrate its effectiveness as a tool for genome-wide insertional mutagenesis in *Plasmodium*. Additionally, in both *P. falciparum* and *P. berghei*, mostly single *piggyBac* insertions are obtained per genome, allowing easy correlation of genotypes to their respective phenotypes [39, 40].

### 4.4. Applications of *piggyBac* Mutagenesis

The most benefiting application of insertional mutagenesis will be the ability to perform forward genetic screens to identify genes contributing to a phenotype of interest. A medium-scale forward genetic screen using approximately 200 *P. falciparum piggyBac* insertional mutants was able to identify several genes and pathways crucial for intraerythrocytic development of the parasite [41]. Although genes essential for *in vitro* intraerythrocytic development could not be identified due to the haploid genome, mutants with reduced growth rates of up to 70% could be isolated. It is also important to realize that many of the genes affected in these severely attenuated mutants might be essential for *in vivo* development, as the host immune system might effectively clear a slowly spreading infection. For example, disruption of the *P. falciparum ccr4-not associated factor 1* (*caf1*) results in severe attenuation of intraerythrocytic growth *in vitro* but, despite multiple attempts, the gene remains refractory to disruption in *P*. *berghei*, suggesting its essentiality *in vivo* [42].

**Figure 3:**
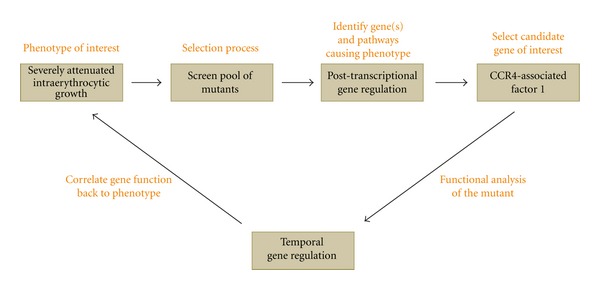
An example of a successful forward genetic approach in *P. falciparum*. A screen for attenuated intraerythrocytic growth indicates the significance of post-transcriptional gene regulation in these stages. Functional characterization of CCR4-Associated Factor 1 has revealed a role in temporal regulation of gene expression in *P. falciparum*, implying its importance in intraerythrocytic development.

Another key finding of this forward genetic study in *P. falciparum* is the significance of nucleic acid binding and nucleic acid metabolism genes in intraerythrocytic development [41]. A majority of genes in this category includes RNA-binding proteins that play a potential role in posttranscriptional gene regulatory mechanisms, thus identifying this process as a high-value target for therapeutic intervention. A follow-up investigation of one of the genes identified in this study substantiates the potential of forward genetic screens in *Plasmodium* (Figure 3). Functional characterization of the *caf1* null mutant reveals a critical role for this gene in temporal gene regulation in *P. falciparum*, and its disruption leads to premature egress from host cells, severely attenuating the intraerythrocytic growth rate [42], providing valuable insights into a largely unknown but critical parasite biological process. Further characterization of this gene regulatory pathway might identify other crucial parasite-specific proteins with great potential as antimalarial drug targets.

While transposon-mediated mutagenesis does not offer a direct way to identify essential genes in the haploid blood stages, its ability to rapidly identify nonessential regions of the genome will be significant in directing our antimalarial drug discovery efforts. Genes that show no reduction in intraerythrocytic growth rate upon disruption can be immediately discarded from consideration as putative intraerythrocytic drug targets. However, their expression and functions in other life cycle stages should be considered for transmission-blocking strategies or for targeting the parasite liver stages.

*piggyBac* offers an excellent vehicle for stable transgene expression in *Plasmodium* without constant drug pressure, which would be required during episomal expression [43–45]. Moreover, insertions in the noncoding regions that do not affect parasite growth can be readily obtained for use in drug-response assays. Expression of parasite proteins as GFP fusions using *piggyBac* allows subcellular localization studies to be performed with ease [44]. Since the insertions are stably maintained in the genome, genes expressed in other parasite life cycle stages can also be studied. DNA sequences up to 7 kb in length including the drug selection cassette can be inserted into the genome quite efficiently; however, longer sequences might require higher levels of transposase for efficient insertion. Most importantly, any parasite line of interest can be readily transformed with *piggyBac* unlike the mycobacteriophage Bxb1-based system, where genomic insertion of phage recombination sites through homologous recombination is a prerequisite to stable expression [46].

 Promoter trapping using transposable elements provided very useful information about gene expression in higher eukaryotes [47, 48]. Promoter trapping has been possible in both *P*. *falciparum* and *P*. *berghei* using *piggyBac* [37, 39]. However, in the current era of large-scale functional genomics, whole-genome transcriptome and other expression studies have provided abundant information about timing and levels of gene expression in *Plasmodium* and the information obtained from promoter trapping might be insignificant. Also, while promoter trapping can help determine active promoters, it cannot identify the exact promoter region, which would still have to be determined through conventional reporter assays.

## 5. Future Directions

While the ability to obtain genomic integrations is tremendously high with *piggyBac* compared to homologous recombination, we are still faced with the challenge of low transfection efficiency in *Plasmodium*. Successful forward genetic screens in other organisms usually involve achieving a “saturation” level of mutagenesis, where every gene in the genome has been perturbed in a single experiment. This might not be possible even in *P. berghei*, which can be transfected at a much higher efficiency than *P. falciparum* and where remobilization of *piggyBac* can be achieved. One should also be wary of the complications that could arise in correlating genotypes to phenotypes in case of uncontrolled remobilization events, and tight regulation of remobilization events will be a necessity for increasing genomic integration events without repeated transfections.

Increasing the mutant pool available for screens is of high priority for the future. Since almost half of the *piggyBac* insertions occur in noncoding regions, they may or may not affect gene expression. The most direct information from forward genetic screens can be obtained by using mutants with disrupted coding sequences, and obtaining as many of them as possible should be one of the prime objectives. Instead of overly speculating about reaching saturation level mutagenesis, the number of experiments needed to disrupt a significant number of genes, and comparison of transformation efficiencies with higher eukaryotes, it might be most beneficial to understand the limitations of *Plasmodium* genetics and make the best use of this system by generating a large number of single insertional mutant clones that could be used in screens. Performing screens in a mixed population of mutants, even if saturation level mutagenesis were possible, would present several challenges in *Plasmodium*. Negative phenotypic screens, such as attenuated growth, failure to produce gametocytes, and inability to infect the mosquito vector, will be of highest interest but extremely difficult to perform and tease out in mixed populations. Even if the phenotype were narrowed down to a set of genes, the lack of robust genetic complementation tools would severely limit further characterization.

Considerable attention must also be given to developing new genetic screens targeted towards the many intriguing features of parasite development and pathogenesis. For example, phenotypic screens for adherence to endothelial cells, which is a critical component of disease pathogenesis, could identify novel mediators of this process. Screens directed towards the development of other parasite life cycle stages, such as gametocytes and sporozoites, should provide novel candidates for transmission-blocking strategies and contribute immensely to disease control and prevention strategies.

**Figure 4:**
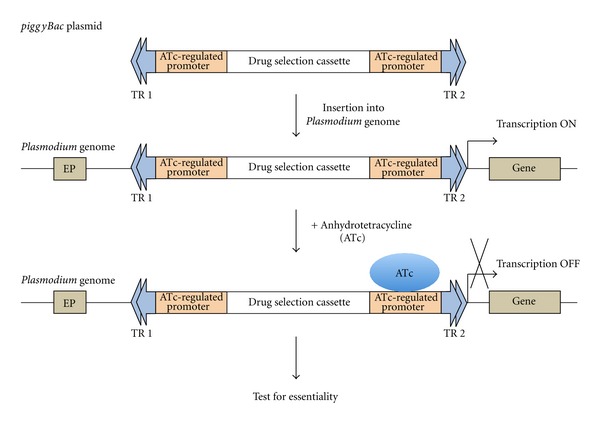
A putative scheme for identifying essential genes in *Plasmodium* using *piggyBac* mutagenesis. The *piggyBac* plasmid containing two anhydrotetracycline-(ATc-) regulated promoters, one near each terminal repeat (TR), can be first inserted into the *Plasmodium* genome. Upon insertion into the 5′ region of a gene, its endogenous promoter (EP) will be replaced by the ATc-regulated promoter. Addition of ATc will block transcription of the gene, allowing the evaluation of its essentiality.

The biggest limitation of insertional mutagenesis in *Plasmodium* is the inability to mutate and identify genes essential for blood stage development, as only the haploid blood stages of the parasite can be transfected. To overcome this deficiency, novel experimental designs that combine the *piggyBac* system with a conditional expression system such as the anhydrotetracycline-(ATc-) regulated system [49], need to be developed. One could envision placing the ATc-regulated promoter in between the *piggyBac* terminal repeats such that, upon insertion into the genome, it replaces an endogenous promoter but is still able to drive the expression of the downstream gene (Figure 4). Addition of ATc would turn off the expression of the downstream gene and its essentiality in blood-stage development could be evaluated. However, several factors would need to be considered for such an approach to work. Since *piggyBac* terminal repeats could be inserted into the genome in either orientation, it might be best to place two ATc-regulated promoters in between the *piggyBac* terminal repeats, one near each terminal repeat (Figure 4). The effectiveness of this system will depend upon its insertion in the 5′ UTR of a gene, and the similarity between the timing of expression from the ATc-regulated promoter and the endogenous promoter. Furthermore, the robustness of the ATc-regulated system would need to be tested as leaky expression through the ATc-regulated promoter would hinder the identification of essential genes. More robust systems using *Plasmodium*-specific factors might be required for tight regulation of gene expression.

 In summary, the introduction of transposon mutagenesis to *Plasmodium* has provided new exploratory avenues to study this important parasite. With further refinement of the system and an increase in the mutant pool, a variety of forward genetic studies should be feasible and provide exciting discoveries about the malaria parasite's enigmatic biology that could be exploited for developing novel antimalarial strategies.
